# Supplementary material for: Vertically stacked monolithic perovskite colour photodetectors
Source: Nature. 2025 Jun 18;642(8068):592–8. doi: 10.1038/s41586-025-09062-3 (PMC12176651; doi:10.1038/s41586-025-09062-3)
Supplement: Supplementary file 1 — Supplementary Information [file 41586_2025_9062_MOESM1_ESM.pdf]

nature portfolio

https://doi.org/10.1038/s41586-025-09062-3

Supplementary information

Vertically stacked monolithic perovskite colour photodetectors

In the format provided by the authors and unedited

Supplementary Information

Vertically stacked monolithic perovskite colour photodetectors

Sergey Tsarev[1,2], Daria Proniakova[1], Xuqi Liu[1], Erfu Wu[1,3], Gebhard J. Matt[1,2], Kostiantyn Sakhatskyi[1,2], Lorenzo L. A. Ferraressi[1,3], Radha Kothandaraman[2], Fan Fu[2], Ivan Shorubalko[3], Sergii Yakunin*[1,2], Maksym V. Kovalenko*[1,2]

[1] Laboratory of Inorganic Chemistry, Department of Chemistry and Applied Biosciences, ETH Zürich, CH-8093 Zürich, Switzerland

[2] Laboratory for Thin Films and Photovoltaics, Empa – Swiss Federal Laboratories for Materials Science and Technology, CH-8600 Dübendorf, Switzerland

[3] Transport at Nanoscale Interfaces Laboratory, Empa – Swiss Federal Laboratories for Materials Science and Technology, CH-8600 Dübendorf, Switzerland

*E-mail: mvkovalenko@ethz.ch; yakunins@ethz.ch

Contents

Supplementary Note 1. Perovskite sensor structure 4

1.1 Red colour detector stack.. 4

1.2 Green colour detector stack 4

1.3 Blue colour detector stack.. 5

Supplementary Table 1. Complete sensor structure 1. 6

Supplementary Table 2. Complete sensor structure 2 7

Supplementary Table 3. Energy Dispersive X-Ray Analysis of films with nominal composition of $MAPbBrI_2$, $CsPbBr_2I$ and $CsPbBr_2Cl$ 8

Supplementary Table 4. Comparative detectivity metrics of broadband perovskite, organic, silicon, and stacked perovskite photodetectors 9

Supplementary Note 2. Alternative technologies promising for monolithic stacked detector fabrication 10

2.1 III-V materials for integration in vertically stacked photodetectors. 10

Supplementary Table 5. Semiconductor compositions suitable for use in colour detector layers amongst III-V semiconductors.. 13

2.2 Organic photodetectors 13

Supplementary Note 3. Procedure for evaluation of colour accuracy of optical sensor........... 17

Supplementary Scheme 1.. 19

Supplementary Table 6. Colour accuracy evaluation ΔE_{Lab} (CIE1976 standard). Comparison of sensors and transformation matrices. 20

Supplementary Table 7. Colour accuracy evaluation ΔE_{Lab} (CIE1976 standard). Comparison of colour reproduction under various illumination conditions (different spectra and colour rendering index, CRI Ra). 20

Supplementary Fig. 1 | Atomic force microscopy images of perovskite layers. 21

Supplementary Fig. 2 | External Quantum Efficiency of a stacked detector with structure 1 .. 22

Supplementary Fig. 3 | 3 dB bandwidth of the B (a), G (b), and R(c) single colour detectors. 22

Supplementary Fig. 4 | Capacitance vs frequency measurements of stacked photodetectors. . 23

Supplementary Fig. 5 | Variation of illumination spectra for evaluation of colour accuracy:.. 23

Supplementary Fig. 6 | Emission spectra of RGB channels emitted by LC-10W diode. 24

Supplementary Fig. 7 | Photos of a. TFT array (G-layer) and b. RGB stacked cross-bar array used in this study... 24

Supplementary Fig. 8 | Linearity of red (a), green (b), blue (c) channels at higher flux for the vertically stacked arrays.. 25

Supplementary Fig. 9 |TFT Image post-processing procedure. 26

Supplementary Fig. 10 | Comparative performance of red detectors with 30 nm and 100 nm bottom gold electrode. 26

Supplementary Fig. 11 | X-ray diffraction patterns and SEM cross-section images of $CsPbBr_2Cl$ films. 27

Supplementary References... 28

Supplementary Note 1. Perovskite sensor structure

1.1 Red colour detector stack

In our approach to developing a stacked perovskite layer, we aimed for methodologies designed for seamless transfer to CMOS-compatible processing. We selected vapour-phase perovskite deposition to facilitate deposition on textured and uneven surfaces, which may be essential for further application on CMOS chips and avoid problems with the dissolution of perovskites by the subsequent layers. We avoided thick dielectric spacers, such as polymer films and parylene, to maintain a relatively thin total sensor thickness. This is vital for future developments of practical sensor arrays, which will require lithographic etching and integrating via connectors onto CMOS chips.

The deposition process begins with dielectric/Au/dielectric electrodes, utilizing MoO_3 as an adhesive layer beneath the gold and as a buffer layer. This reflective Au electrode was used to enhance the light absorption in the red detection layer. We discovered that keeping the gold layer thin (30 nm) was essential for achieving leak-free detectors, as devices with 100 nm gold layers exhibited significantly higher leakage (Supplementary Fig. 10). In general, these stacked structures are very demanding of having sharp-edge-free bottom electrodes. We chose $MAPbI_2Br$ for its ease of use and reproducibility as the perovskite composition. The structure was completed with a C60/ZnO/ITO layer, employing ZnO as a protective layer to mitigate sputter damage on the perovskite/ETL stack. Continuing the fabrication, we deposited dielectric stacks. It was noted that while the main ITO electrode surface was sufficiently protective against perovskite decomposition during processing, areas not covered by ITO (deposited through a shadow mask) might react with subsequent perovskite layer stacks. Initially, a combination of SnO_2 and SiO_2 was included as protective and dielectric layers. The SnO_2 layer (deposited with pulsed DC sputtering) was later selected due to observed degradation and bleaching of perovskite when SiO_2 was sputtered (RF) directly onto areas of the perovskite substrate without ITO coverage.

1.2 Green colour detector stack

The green stack's fabrication commenced with the deposition of the ITO electrode and a SnO_2/C60 ETL stack. We selected $CsPbBr_2I$ as a stable inorganic perovskite for the perovskite layer. We encountered low batch-to-batch reproducibility while experimenting with the thermally evaporated $MAPbBr_{2.5}I_{0.5}$ composition. Given the entire sensor fabrication process could extend

up to 3-4 weeks, maintaining high reproducibility was deemed crucial, leading to a focus on inorganic perovskites. A composite HTL comprising TCTA/MoO_3 and an additional AZO nanocrystal buffer layer was used to minimize the sputtering damage on the green cell. The TCTA layer was chosen to maintain vapour deposition for the entire stack and its high glass transition temperature[1], crucial for the thermal stability of the cell upon deposition of the third stack.

1.3 Blue colour detector stack

The fabrication of the blue stack began with the deposition of a 2PACz and NiO composite layer. The initial 2PACz coating provided a hole-selective interface coating for the edges of the bottom ITO electrode, addressing the potential nonuniform coating of NiO NC coating on the sharp electrode edges. The $CsPbBr_2Cl$ layer was used as the absorber layer due to its high stability. Although we experimented with $MAPbBr_{1.5}Cl_{1.5}$ as an absorber layer, we could not achieve an EQE higher than 20% with this composition. Notably, we achieved an 81% EQE with high-performance solution-processed $FAPbBr_{1.5}Cl_{1.5}$ detectors. However, the evaporation of FABr or FACl precursors in our PVD chamber proved challenging. To maintain consistency with PVD-processed stacks, we decided against incorporating any solution-processed perovskite layers in the stack, further complicated by issues of perovskite dissolution in the bottom layers when a solution-based composition was used.

Supplementary Table 1. Complete sensor structure 1. Layers are deposited in the order from layer 1, beginning with a 25x25 mm soda lime glass substrate.

	Layer	Nominal thickness (nm)	Deposition method	Role
1	MoO_3	15	Thermal evaporation	Adhesion
2	Gold	30	Thermal evaporation	Electrode
3	MoO_3	30	Thermal evaporation	Buffer
4	2PACz	<2	Spin coating	Hole-selective
5	$MAPbBrI_2$	450	Thermal evaporation	Absorber
6	C60	20	Thermal evaporation	Electron-transport
7	ZnO	40	Spin coating	Buffer
8	ITO	180	Sputtering	Electrode
9	SnO_2	40	Sputtering	Protective
10	SiO_2	200	Sputtering	Dielectric
11	ITO	180	Sputtering	Electrode
12	SnO_2	40	Sputtering	Electron-transport
13	C60	5	Thermal evaporation	Electron-transport
14	$CsPbBr_2I$	300	Thermal evaporation	Absorber
15	TCTA	15	Thermal evaporation	Hole-transport
16	MoO_3	30	Thermal evaporation	Buffer
17	AZO	40	Spin coating	Buffer
18	ITO	140	Sputtering	Electrode
19	SnO_2	40	Sputtering	Protective
20	SiO_2	200	Sputtering	Dielectric
21	ITO	100	Sputtering	Electrode
22	2PACz	<2	Spin coating	Hole selective
23	NiO	30	Spin coating	Hole transport
24	TCTA	5	Thermal evaporation	Hole transport
25	$CsPbBr_2Cl$	200	Thermal evaporation	Absorber
26	PCBM	~40	Spin coating	Electron-transport
27	C60	5	Thermal evaporation	Electron-transport
28	ZnO	40	Spin coating	Buffer
29	ITO	120	Sputtering	Electrode

Supplementary Table 2. Complete sensor structure 2. Layers are deposited in the order from layer 1, beginning with a 25x25 mm soda lime glass substrate.

	Layer	Nominal thickness (nm)	Deposition method	Role
1	MoO_3	15	Thermal evaporation	Adhesion
2	Gold	30	Thermal evaporation	Electrode
3	MoO_3	30	Thermal evaporation	Buffer
4	2PACz	<2	Spin coating	Hole-selective
5	$MAPbBrI_2$	450	Thermal evaporation	Absorber
6	C60	40	Thermal evaporation	Electron-transport
7	ZnO	40	Spin coating	Buffer
8	SnO_2	40	ALD	Buffer
9	ITO	180	Sputtering	Electrode
10	SnO_2	40	Sputtering	Electron-transport
11	C60	5	Thermal evaporation	Electron-transport
12	$CsPbBr_2I$	300	Thermal evaporation	Absorber
13	TCTA	15	Thermal evaporation	Hole-transport
14	MoO_3	30	Thermal evaporation	Buffer
15	ITO	100	Sputtering	Electrode
16	2PACz	<2	Spin coating	Hole selective
17	NiO	30	Spin coating	Hole transport
18	TCTA	5	Thermal evaporation	Hole transport
19	$CsPbBr_2Cl$	400	Thermal evaporation	Absorber
20	C60	20	Thermal evaporation	Electron-transport
21	BCP	8	Thermal evaporation	Buffer
22	Ag	1	Thermal evaporation	Adhesive layer
23	ZnO	40	Spin coating	Buffer
24	ITO	120	Sputtering	Electrode

In **Structure 2**, we implemented the following modifications: a buffer layer of ALD SnO_2 was added to enhance the protection of the red cell from the layers above. It was determined that using a conformal protection layer avoids the need for a dielectric layer, which, in turn, contributed to an increase in the EQE of the red cell. For the green cell, it was found that incorporating an AZO nanocrystal buffer layer significantly compromised the environmental stability of the sensors, which would be crucial for further integration into CMOS as most of the conventional clean-room

processes occur under ambient conditions. Furthermore, we removed dielectrics in the stack to minimize optical losses and simplify the fabrication.

The blue cell structure underwent a complete redesign, replacing the $CsPbBr_2Cl$ uniform film with a gradient-grown film, utilizing the deposition of CsCl at a higher rate initially. These films exhibited a significantly higher EQE than those with uniform growth, enabling us to achieve a peak EQE of 54% in a top-illuminated structure. SEM cross-sections indicated the formation of a bilayer, likely with Cs-rich and Cs-poor regions, while XRD reveals broader peaks and mixed (100) and (110) orientations compared to a prevailing (110) texture in uniformly evaporated films (Supplementary Fig. 11). Furthermore, EDX indicated the formation of a Cs-rich film for the gradient-grown film, while a lead-rich composition was observed for the uniform film, despite nominally equal stoichiometry calculated by components evaporation rates. This bilayer structure likely improves vertical charge transport, facilitating charge extraction, possibly via the formation of p - and n - doped regions within the film[2]. As for the ETL, a combination of C60/BCP/Ag/ZnO layers was found to outperform pure C60/ZnO, presumably due to enhanced hole blocking and improved protection of the perovskite layer from sputter damage.

Supplementary Table 3. Energy Dispersive X-Ray Analysis of films with nominal composition of $MAPbBrI_2$, $CsPbBr_2I$ and $CsPbBr_2Cl$

Nominal film composition	Cs, at %	Pb, at %	Br, at %	I, at %	Cl, at %
$CsPbBr_2Cl$ - uniform	17.2	21.6	30.4	N/A	30.7
$CsPbBr_2Cl$ -gradient	19.7	16.5	32.9	N/A	30.8
$CsPbBr_2I$	19.8	22.0	37.2	21.6	N/A
$MAPbBr_2I$	N/A	25.0	20.3	54.7	N/A

1Supplementary Table 4. Comparative detectivity metrics of broadband perovskite, organic, silicon, and stacked perovskite photodetectors

**Detector**	**D (Jones)**	**Reference**
Stacked - blue	2×10^{10}	Our work
Stacked - green	8.6×10^{10}	Our work
Stacked - red	1.5×10^{11}	Our work
State-of-the-art broadband perovskite detectors		
$PTAA/MAPbI_3/C_{60}$	7.8×10^{12}	[3]
TiO_2/Al_2O_3/PCBM/$MAPbI_3$/Spiro/Au	4×10^{12}	[4]
$NiO_x:PbI_2/MAPbI_3/C_{60}$	4.0×10^{12}	[5]
Silicon detectors		
FDS1010 (Thorlabs)	4.8×10^{12} at $1 cm^2$ (20 V)	[6]
FDS100 (Thorlabs)	3×10^{13} at 13 mm^2 (5 V)	[6]
818-BB-45 (Hamamatsu)	1.1×10^{12} at 1.1 mm^2	[7]
Organic photodetectors		
$PCDTBT:PC_{71}BM$	1.8×10^{12} (− 1 V)	[8]
P3HT:PTB7: $PC_{71}BM$	1.1×10^{12} (0 V)	[9]
$PBTTT:PC_{61}BM$	3.6×10^{12} (0 V)	[10]

Supplementary Note 2. Alternative technologies promising for monolithic stacked detector fabrication

While perovskite absorber materials hold potential for monolithic stacked detector fabrication, it is important to note that this technology is not restricted to specific absorber materials. Any photosensitive materials possessing appropriate bandgap and transparency characteristics can potentially serve as a viable option for colour sensors. Furthermore, different classes of materials can potentially be combined, offering greater flexibility in the design and characteristics of the sensor's active layers. Alongside perovskite, we consider two classes of materials with tuneable bandgaps that hold promise for vertical stacking: III-V semiconductors and bulk heterojunction organic semiconductors. These material families have been extensively employed in tandem solar cells, which share a fundamentally similar architectural framework with stacked diode sensors. This note discusses existing technologies of III-V and organic semiconductors within the context of stacked detector applications.

2.1 III-V materials for integration in vertically stacked photodetectors.

Epitaxially grown III-V alloys offer superior optoelectronic materials characterized by a wide range of tunable bandgaps, as evidenced by numerous ultra-high-efficiency multijunction solar cells employed in concentrator and space applications[11,12]. Generally, the primary concern associated with III-V semiconductors is their cost, rendering them primarily suitable for niche applications that demand high performance and tolerate higher costs, such as space energy sources[13], ultra-high bandwidth detectors[14,15], and concentrator solar cells[11,16,17]. Notably, recent advancements, particularly the adoption of an ultrasonic lift-off technique, have substantially mitigated costs by enabling substrate reuse, achieving a cost reduction of 4-6 times compared to traditional molecular beam epitaxy (MBE) growth techniques[18]. However, despite these cost reductions, lattice matching between film and substrate remains a critical factor in epitaxial growth processes, also influencing the performance of resulting devices[11,12,19]. Here, we will address the primary III-V materials suitable for detecting colours within the visible spectrum, particularly those that align with the RGB colour range. The summary of these findings is shown in Supplementary Table 5

2.1.1. Gallium-indium phosphide based materials

$Ga_xIn_{1-x}P$ is utilised in the top and middle junction cells for terrestrial concentrator applications due to its tuneable bandgap ranging from approximately 1.68 to 1.93 eV[19,20]. This range of

bandgaps renders it suitable for use in red colour detector pixels. The material is commonly employed as a top cell component in tandem multijunction cells yielding 47.6% efficient solar cells in 2022[17]. Typically, the growth of this material is confined to lattice-matching substrates such as GaAs wafers, as lattice mismatch can lead to a decrease in performance due to a high density of lattice dislocations at the interface with the substrate[21]. It has been noted that obtaining high-quality $Ga_xIn_{1-x}P$ is comparatively easier than $Al_xGa_{1-x}As$ due to the latter's strong affinity for the formation of Al-O defects during growth[22].

$Al_xGa_yIn_{1-x-y}P$ is employed in multijunction devices as a top cell component of tandem multijunction cells, typically grown directly on GaAs wafers or films interconnected via a tunnel junction, as reported in a recent paper highlighting solar cells with the second-highest performance at 47.1[11] from NREL. If we extrapolate the geometry of this top-performing multijunction cell to the photodetector context, then the top two junctions, with bandgaps of 1.7 eV ($Al_xGa_{1-x}As$) and 2.1 eV, can potentially be utilised for red and green colour detection, respectively, based on light absorption considerations.

However, the majority of reported phosphide and arsenide materials, which are direct bandgap semiconductors, typically have bandgaps reaching up to 2.38 eV, slightly below the approximately 2.5 eV bandgap[23] required to maintain selectivity to blue light. Given that most absorbers in tandem solar cells do not utilize such wide bandgap materials, it becomes necessary to integrate a different material specifically tailored for blue light detection.

2.1.2. Gallium nitride and indium gallium nitride-based materials

GaN-based nitride semiconductors have significantly advanced the development of optoelectronic devices, particularly laser diodes, LEDs, and UV photodetectors. The bandgap of gallium nitride (GaN) can be tuned by incorporating indium nitride (InN), resulting in the formation of InxGa$_{1-}$xN semiconductors. This modification allows for a variable bandgap that extends from 3.42 eV, characteristic of pure GaN, down to 1.7 eV. Furthermore, it is suggested that the bandgap could be reduced to as low as 0.64 eV with the use of pure InN[24,25]. However, achieving uniform bandgap tunability is challenging due to the difference in lattice spacing between InN and GaN[26]. The lattice mismatch between these materials presents obstacles in the growth of mixed $In_xGa_{1-x}N$ films, although this challenge can be mitigated by utilizing GaN or AlN buffer layers as templates[27]. Furthermore, InxGa1-xN tends to exhibit n-type semiconductivity and must be doped to form p-n

junctions for photodiodes. This process is hindered by the necessity to generate a high density of defects for p-doping to compensate for an excess of electrons in the material[28]. Nonetheless, $In_xGa_{1-x}N$ has emerged as a promising material for blue light detection in photodetectors[14,29,30], and micro-detector arrays[15], demonstrating high responsivity (.29 A/W at 400nm[15]) and low dark current detectors. Moreover, direct integration of $In_xGa_{1-x}N$ on Si via a buffer GaN layer has been demonstrated[14]. Additionally, there have been successful attempts towards integrating $Al_xGa_yIn_{1-x-y}P$ and $In_xGa_{1-x}N$ in dual-band LEDs, showcasing the potential for their successful incorporation into multiband colour detectors[31].

When it comes to detecting green light, it has been observed that as the band gap decreases, $In_xGa_{1-x}N$ experiences greater susceptibility to phase separation and high defect concentrations, resulting in poor colour selectivity and increased leakage in devices [32]. Reports on green-light detection using $In_xGa_{1-x}N$ are scarce[32], potentially due to its performance significantly lagging behind silicon performance with optical filters. Nevertheless, there are reports of existing multijunction solar cells employing $In_xGa_{1-x}N$ in conjunction with silicon, offering promise for the potential integration of this material as a middle layer in Si (red)/$In_xGa_{1-x}N$ (green)/ $In_yGa_{1-y}N$ (blue) configurations[33]. However, thus far, only a 60% external quantum efficiency (EQE) has been demonstrated for $In_xGa_{1-x}N$[33], and research in this field remains relatively inactive.

2.1.3. Summary for III-V photodetectors

To sum up, a potentially viable sensor multistack comprising only III-V semiconductors as absorbers would entail lattice-matched $Ga_xIn_{1-x}P$ or $Al_xGa_{1-x}As$ for red and $Al_xGa_yIn_{1-x-y}P$ for green detection layers, respectively, integrated with an $In_xGa_{1-x}N$ layer via a GaN buffer layer. Achieving a sufficiently low degree of lattice mismatch between the nitride and phosphide/arsenide components would be crucial, possibly necessitating separate deposition of blue and R-G detectors and employing a lift-off transfer process for the former. However, even if successful, questions remain regarding how to pattern and make electrical contacts to this structure. For this purpose, one can deposit a transparent conductive oxide electrode, which, however, will interfere with the epitaxial growth of absorber layers on top of this layer. Alternatively, if heavily doped contacts are used, it is not apparent how feasible is to dope them and electrically separate them from the main stack to obtain a low-leakage device. Thus, the extremely intricate and costly fabrication procedure may be difficult to justify for commercial consumer-grade applications.

Nevertheless, if successful transfer or growth of the blue cell onto a lattice-matched R-G cell with proper alignment, coupled with the successful implementation of contacts, is achieved, these detectors are anticipated to surpass perovskites in terms of both performance and stability.

Supplementary Table 5. Semiconductor compositions suitable for use in colour detector layers amongst III-V semiconductors.

Composition	Target colour	Band gap, eV	Substrate
$Ga_xIn_{x-1}P$	R, G	1.83-1.93[20],1.68-1.88[19]; 2.2-1.4 (pure InP)[34]	GaAs wafer[20]
AlxGa1-xAs	R, G	1.42-2.16eV[35]	GaAs wafer[35]
$AlxGayIn_{1-x-y}P$	G	2.1[12]	GaAs
AlxGa1-xP	R, G	2.33-1.4[34]; 2.31[36]	GaAs
InxGa1-xN	B	2.8-1.7[37], 0.77-3.4[26], 3.5-1.7[38]	GaN, or GaN buffer

2.2 Organic photodetectors

In contrast to III-V semiconductors and perovskites, organic semiconductors have received much attention as highly suitable materials for vertically stacked colour sensors, primarily due to their exceptional spectral tunability. This tunability is achievable through molecular engineering, facilitating the development of photodetectors that are inherently sensitive to various spectral regions. Additionally, organic photodetectors are attractive for their manufacturability using low-temperature, solution-based techniques, such as printing[39,40] and spincoating[41,42], especially on flexible plastic substrates.

However, the fabrication of stacked organic photodetectors presents significant challenges. These include sputter damage incurred during the deposition of transparent conductive oxides[43-45], and the requirement to select orthogonal solvent mixtures for the deposition of colour-sensitive layers to prevent dissolution of underlying layers[41,46,47]. In the case of thermally evaporated detectors, fabricating highly efficient devices poses significant challenges, primarily due to the inherent

difficulties in forming bulk heterojunctions using a vapour deposition process[48,49]. Moreover, there is a notable scarcity of high-performance blue light detectors in the literature[50].

Despite these challenges, there have been successful demonstrations of integrating organic semiconductors with silicon chips,[42,51-53] including the development of stacked organic sensors[41,54-56]. There also exist reports about mechanically stacked triple-colour organic detectors[57] of a single instance of a vertically stacked triple-colour sensor using thin metal electrodes[58], likely utilised due to the technological difficulties associated with applying sputtered electrodes in organic photodetectors. This note seeks to evaluate these challenges and explore why a fully functional triple organic photodiode has yet to be reported, aiming to provide insights into the limitations and potential breakthroughs in this field.

2.2.1 Colour selectivity of organic sensors

The colour selectivity of organic sensors, particularly in the blue light spectrum, remains a challenging matter in the field of organic electronics. While numerous studies have focused on developing green[59-61] and broadband[9,62,63] organic detectors, the availability of organic materials suitable for blue light detection is comparatively limited. The most efficient blue detectors typically employ mixtures of C_{60} or C_{70} fullerene derivatives and donor molecules, such as rubrene[50,64] or PffBT4T-2OD(Poly[(5,6-difluoro-2,1,3-benzothiadiazol-4,7-diyl)-alt-(3,3’’’-di(2-octyldodecyl)-2,2’;5’,2’’;5’’,2’’’-quaterthiophen-5,5’’’-diyl)])[65]. However, the absorption spectrum of C60 does not align well with the blue spectral region, leading to suboptimal colour selectivity in such devices. For instance, in stacked sensors with blue and green channels, a 20% response to green light (500-600nm) was observed from the blue channel, likely due to the optical absorbance from the $PC_{61}BM$ ([6,6]-phenyl-C61-butyric acid methyl ester) layer[41]. An alternative strategy involves the use of homojunctions with blue-sensitive chromophores. A notable example of colour selective mechanically stacked 3-layer sensor used Co-TTP (tetraphenylporphyrin) which exhibits an absorption maximum at 430 nm. This peak, however, falls short of covering the entire visible blue region, leading to colour selectivity that is inferior to that of modern colour-filter arrays[66]. Additionally, DPh-DNTT (diphenyl-dinaphtho[2,3-b:2′,3′-f]-thieno[3,2-b] thiophene) has been explored for blue light sensitivity, aiming to minimize the blue cell's response to green and red light, with an absorption edge at around 520 nm[55]. Despite these efforts, high levels of colour crosstalk for blue light have been observed, attributed to the incomplete absorption

by the blue-sensitive layer. Other notable approaches have included the use of pure F8BT (poly(9,9-di-n-octylfluorenyl-2,7-diyl)-co-1,4-benzo-(2,1,3)-thiadiazole)[67] or F8BT doped with DMTPS (1,1-dimethyl-2,3,4,5-tetraphenylsilole)[26]. Overall, aligning the absorption characteristics of organic materials with their electronic properties sufficient for high-performance detectors may not be as straightforward as with perovskites. This complexity suggests that challenges in developing stacked organic sensors, particularly those with precise colour selectivity, may impede rapid progress in this area.

2.2.2 Transparent electrodes and sputter damage in organic photodetectors

Sputtering is a plasma-assisted, physical vapour deposition method that involves the bombardment of a target material by high-energy ions followed by the deposition of the ejected particles onto a substrate. The sputtering processes are widely used for the fabrication of transparent conductive oxide electrodes that possess superior transparency and stability compared to thin metal electrodes[68]. However, a significant drawback of this technique is the sputtering damage inflicted on the underlying layers, attributable to the bombardment with highly energetic ions[44,68,69]. This issue is of particular concern for organic semiconductors, which are vulnerable to oxygen and are physically soft, rendering them sensitive to such damage[44]. As a result, many organic devices traditionally opt for metal or semi-transparent metal electrodes, despite the compromise to the optical efficiency of stacked devices[58].

Recently, a growing interest has been focused on semi-transparent organic solar cells and photodetectors that incorporate transparent conductive oxides deposited atop the organic layer[45,56]. This interest is likely spurred by advancements in tandem perovskite solar cells, which encounter similar challenges related to sputter damage[70,71]. Additionally, less damaging transparent conductive oxide deposition methods can be used such as eclipse pulsed laser deposition, have demonstrated compatibility with organic solar cells (OSC), offering performance on par with metallic references [72]. Nonetheless, these methods are often expensive and require sophisticated equipment.

2.2.3 Summary of organic photodetectors

Unlike perovskites, where bandgap tunability is achieved through halide mixing, tuning the bandgap in organic materials requires altering the molecular composition, which can impose significant challenges on the fabrication of multiband detectors. The limited availability of high-

performance vapour-deposited organic photosensitive films necessitates reliance on solution-processed detectors, introducing challenges related to solvent compatibility between layers. Moreover, the broad absorption profile of organic detectors may not be as advantageous for machine vision applications when compared to perovskites, as discussed in Supplementary Note 3, suggesting no direct benefit in terms of colour accuracy enhancement over existing colour filter arrays. On the other hand, organic materials do not suffer from ion migration[73], a significant concern for the stability of perovskite detectors. Despite the challenges, organic materials remain promising candidates for the development of stacked detectors, and their performance warrants comparison with perovskite technology as an alternative approach to multicolour pixel conception. Moreover, an important opportunity might arise from the monolithic integration of organic and perovskite layers in one architecture, facilitated by the orthogonal solubility of perovskites and organic semiconductors in polar and non-polar solvents.

Supplementary Note 3. Procedure for evaluation of colour accuracy of optical sensor

The colour accuracy of the sensor has been tested under laboratory conditions that closely mimic the natural light reception processes, affording a quantitative evaluation. We used an optical stage (Fig. 3 a) where we positioned a light source, D50-equivalent with emission spectrum $W(\lambda)$ presented in Fig. 3 b (broadband white LED from LCFOCUS, FS-504, 10W, 380-780 nm), illuminating a translation stage with Macbeth ColorChecker with patches chart representing a standard colour palette (the individual patches (m,n) are represented by albedo spectra $A_{m,n}(\lambda)$). The chart was masked with a black opaque stencil so that solely individual patches were illuminated. The light scattered from the ColorChecker patch is collimated and focused on the perovskite stacked photodetectors. The photodetectors were operated under 0 V bias and the photocurrents from all three channels were measured in the form of vectors with values of so-called device-dependent raw-RGB signals for each (m,n) of 24 patches of ColorChecker as presented in Fig. 3a (in the experiment with photocurrent measurement we subtracted dark currents as the setup received some amount of stray light from the sample stage). In parallel, we evaluated (step (1) on Supplementary Scheme 1) raw-RGB signals $P_{i,m,n}$ ($i=R, G, B$) using the integral of a product for experimentally recorded EQE spectra of individual channels of the monolithically stacked device as well as measured spectra of absorption $A_{n,m}$ for ColorChecker and various illumination $W(\lambda)$: white LED lamp with D_{50} spectrum, luminescent lamp, daylight sky, and low-R_a RGB LED lamp:

$$P_{i,m,n} = \int_{400nm}^{800nm} W(\lambda) A_{m,n}(\lambda)\, \mathrm{EQE_i}(\lambda) d\lambda \qquad \text{(S1).}$$

Generally, both methods are similar; however, the latter approach enabled a procedure for direct comparison of sensors made by different technologies (CFA and Foveon) based on their spectral EQEs obtained from the literature (Fig. 1c and f). Further, the colour characteristics of the light coming to the sensor (*i.e.* reference colour) can be described as step (2), see Supplementary Scheme 1, in human-specific space as a colour coordinate vector $\begin{pmatrix} X \\ Y \\ Z \end{pmatrix}_{m,n}$ for each m,n path *via* spectral integral:

$$\begin{pmatrix} X \\ Y \\ Z \end{pmatrix}_{m,n} = \int_{400nm}^{800nm} W(\lambda) A_{m,n}(\lambda) \begin{pmatrix} x(\lambda) \\ y(\lambda) \\ z(\lambda) \end{pmatrix}_{m,n} d\lambda \qquad \text{(S2).}$$

where x,y,z are CIE *XYZ* standard observer colour-matching functions. Alternatively, the colour parameters specified by the ColorChecker producer can be used, however, considering larger possible errors due to variations of the measurement setups.

Raw, device-dependent RGB values must be converted (3) into a device-independent colour space (*e.g.*, *XYZ*, *sRGB*, or *Lab*). We selected *sRGB* because it offers a straightforward conversion procedure and clearly demonstrates the technological advantages. This conversion depends on a device-specific transformation matrix (*i.e.* a camera profile), whose determination can be quite sophisticated, often requiring multi-parameter optimisation[74] or neural network[75] approaches and are specific science by itself, the field where we are not fully competent and try to omit in this work. Here, we generally use either a unity diagonal matrix M_1 (especially useful for direct comparisons between different technologies) or near-diagonal matrices M_{corr} to demonstrate the possibility of achieving a higher level of colour accuracy. The merit of how the matrix is close to the diagonal form (as smaller to 0 as closer) can be expressed by the Frobenius norm (normalized ratio for off-diagonal and diagonal matrix entries):

$$F(M_{corr}) = \frac{\sum_{i\neq j}|a_{i,j}|^2}{\sum_{i,j}|a_{i,j}|^2}, \quad \text{(S3)}$$

Where $a_{i,j}$ are elements of matrix M. Notably, the closer the transformation matrix is to the diagonal form, the better the signal-to-noise characteristics and the more reliable the camera's performance under various illumination conditions. Further, the signals are subject to gamma correction ($x=y^{\gamma}$) and white balancing (3). Then device-independent *sRGB* values obtained in the experiment were used to visualize results and further for *sRGB*>*Lab*-conversion (4) to *Lab* colour space that is particularly convenient for evaluation or colour errors. That transformation is standard and can be found elsewhere, we used an online handbook[76] for reference and verification. Similarly, reference *XYZ* characteristics of specific ColorChecker are *XYZ*>*Lab*-converted (5) to the same Lab space for evaluation of averaged colour error (6):

$$\Delta E_{Lab\ CIE1976} = \sum_{m,n}\sqrt{\left(L_{m,n} - L_{ref_{m,n}}\right)^2 + \left(a_{m,n} - a_{ref_{m,n}}\right)^2 + \left(b_{m,n} - b_{ref_{m,n}}\right)^2} \quad \text{(S4)}$$

The ΔE_{Lab} values are averaged over all (n,m) patches of the ColorChecker chart and are presented in Supplementary Tables 6 and 7.

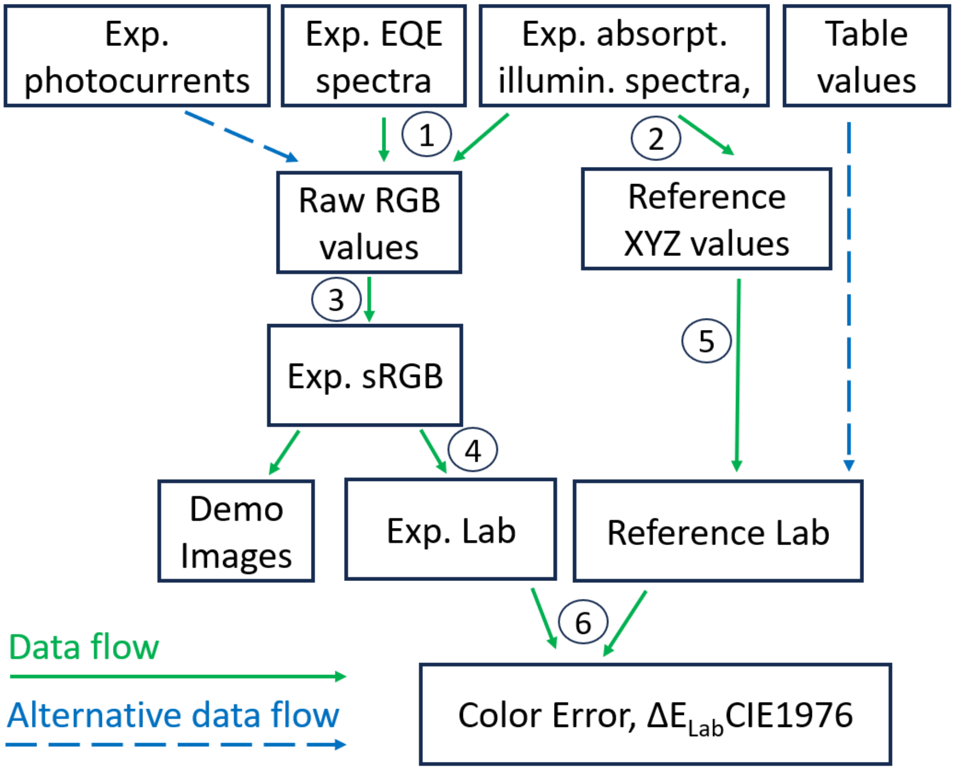

Supplementary Scheme 1 | Mathematical procedure for colour accuracy evaluation: 1. Calculation of photocurrent values from measured EQE, illumination, and absorption spectra, see the equation (S1); **2.** Calculation of reference *XYZ* values from measured illumination and absorption spectra with CIE *XYZ* standard observer colour matching functions, see the equation (S2); **3**. Device-specific matrix transformation and gamma-correction; **4**. *sRGB>Lab* conversion for experimental values; **5**. *XYZ>Lab* conversion for reference values; **6**. Colour error ΔE_{Lab} CIE1976 evaluation according to equation (S4).

Supplementary Table 6. Colour accuracy evaluation ΔE_{Lab} (CIE1976 standard). Comparison of sensors and transformation matrices.

**Sensor, evaluation conditions**	**ΔE_{Lab}**		
	with M_1*	with M_{corr}**	corresp. $F(M_{corr})$**
CFA, Sony IMX249, calculated from literature data for EQE spectra	16.9	7.4	0.28
Foveon sensor, calculated from literature data for EQE spectra, gamma correction index γ=1/2.4	27.5	12.8	0.56
Stacked perovskite sensor, single pixel 4x4 mm^2, calculated from measured EQE spectra	11.2	3.8	0.18
Stacked perovskite sensor, single pixel 4x4 mm^2, photocurrents measured in the experiment (with additional dark subtraction step for raw date and gamma correction index γ=1/2)	10.5	7.6	0.11

* M_1 – diagonal unity matrix,

** M_{corr} near-to-diagonal matrices raw data, optimised for each sensor and their corresponding Frobenius norms $F(M_{corr})$ (normalized ratios for off-diagonal and diagonal matrix entries).

Supplementary Table 7. Colour accuracy evaluation ΔE_{Lab} (CIE1976 standard). Comparison of colour reproduction under various illumination conditions (different spectra and colour rendering index, CRI Ra).

**Illumination conditions**	**CRI, Ra**	**Silicon CFA Sony IMX249**	**Foveon (Stacked-type Si)**	**Stacked Perovskite**
White light LED, D50	98	7.4	12.8	3.8
Daylight sky	97	6.3	20.8	8.5
Luminescent lamp	82	7.3	10.8	3.5
RGB LED lamp	60	6.6	9.65	6.2

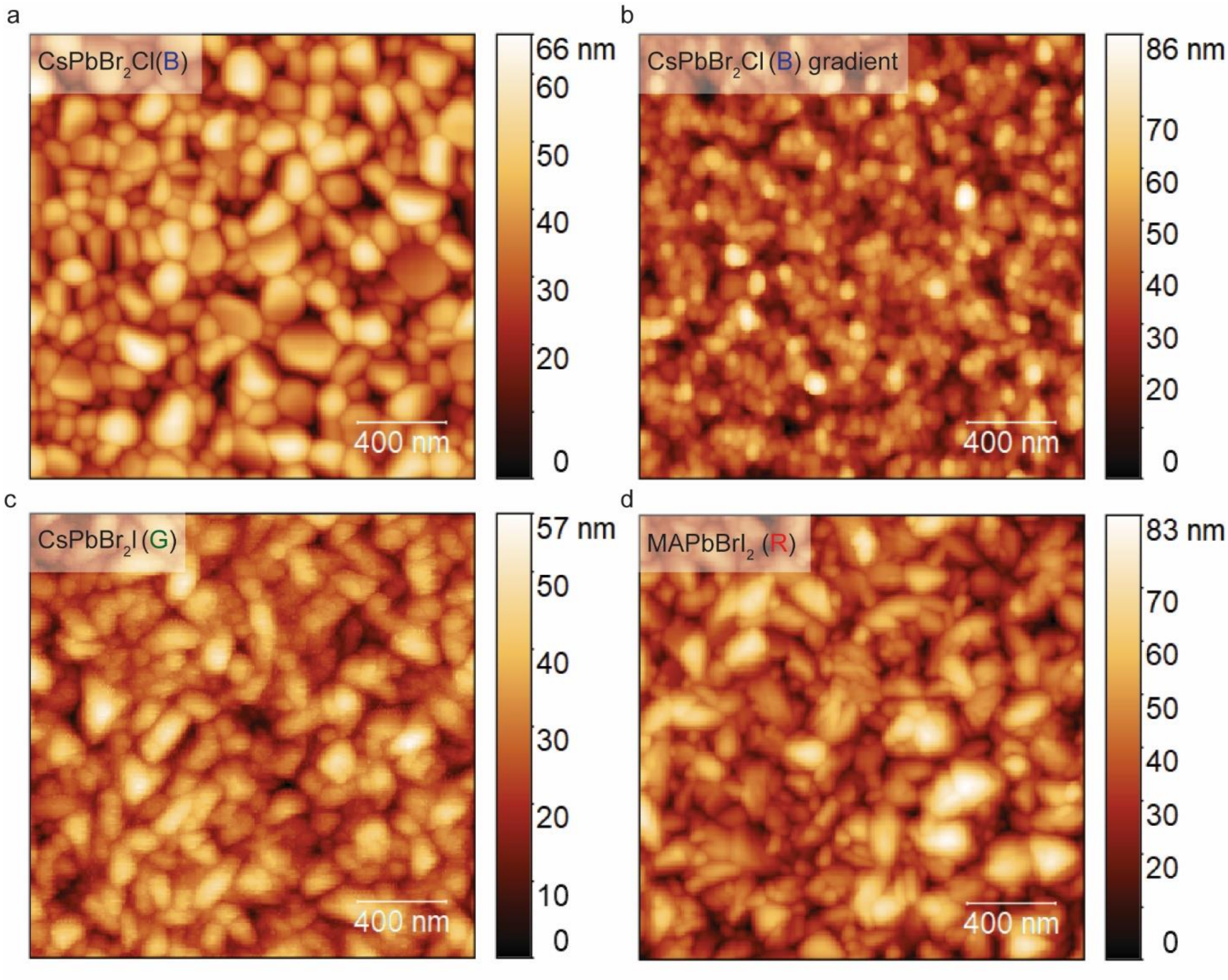

Supplementary Fig. 1 | Atomic force microscopy images of perovskite layers. a, b $CsPbBr_2Cl$ (B) **c.** $CsPbBr_2I$ (G), **d**. $MAPbBr_2I$ (R), in normal and gradient configurations, were deposited on substrates that mimic the configuration of a single-colour detector stack, specifically ITO/ETL for G detectors, or ITO/HTL for R and B detectors. Scans were collected in tapping mode in normal and gradient configurations and deposited on substrates that mimic the configuration of a single-colour detector stack, specifically ITO/ETL for G detectors, or ITO/HTL for R and B detectors.

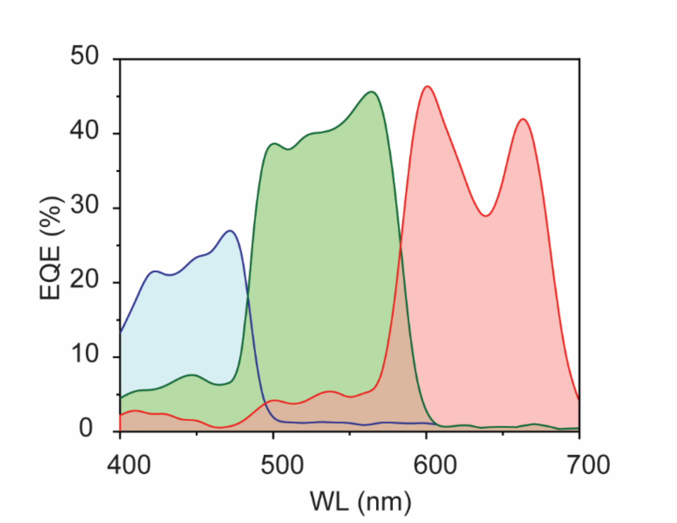

Supplementary Fig. 2 | External Quantum Efficiency of a stacked detector with structure 1. The 4-terminal structure contained $CsPbBr_2Cl$ (B) $CsPbBr_2I$ (G) $MAPbBrI_2$ (R) based detectors sandwiched between charge transport layers. R and G layers are separated with SnO_2/SiO_2 dielectric layer.

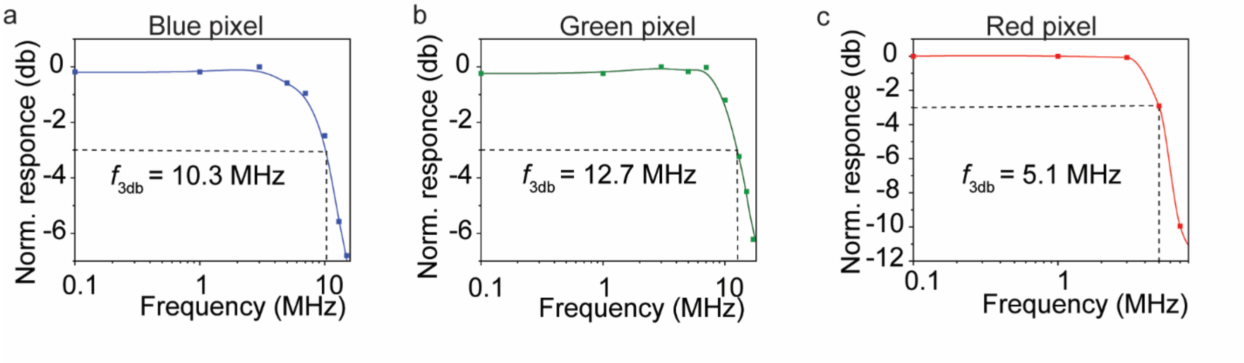

Supplementary Fig. 3 | 3 dB bandwidth of the B (a), G (b), and R(c) single colour detectors. For these measurements, we fabricated smaller area (20x200µm) photodetectors to avoid RC-limited response. Additionally, we replaced the ITO layer with thin (30 nm) gold. The detector responses were collected using a pico-second blue laser with a variable repetition rate.

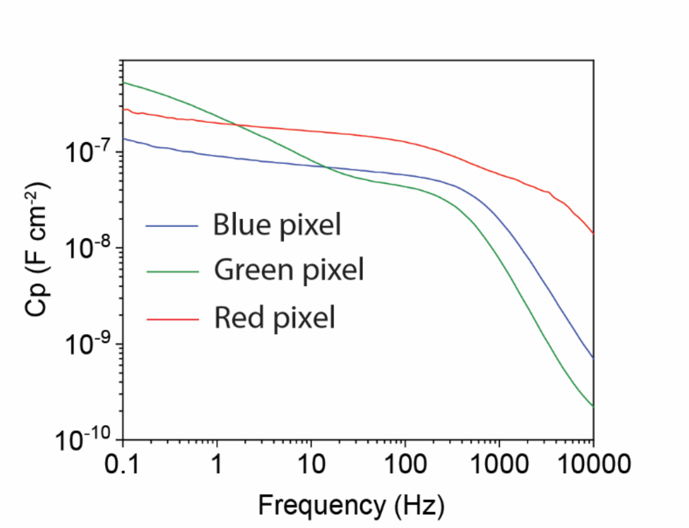

Supplementary Fig. 4 | Capacitance vs frequency measurements of stacked photodetectors. Capacitance was measured using impedance spectroscopy measurements and structure 1 pixel with 0.06 mm2 (B)| 0.09 mm2 (G)| 0.06 mm2 (B) detector area. The measurements were done under dark conditions.

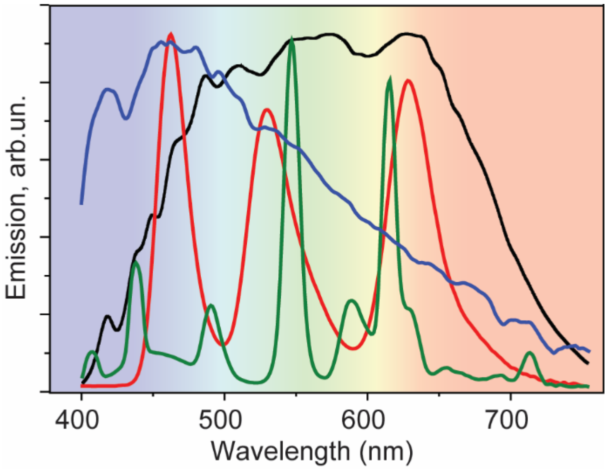

Supplementary Fig. 5 | Variation of illumination spectra for evaluation of colour accuracy: white light LED, D50 (black); daylight sky (blue); luminescent lamp (green); RGB LED lamp (red).

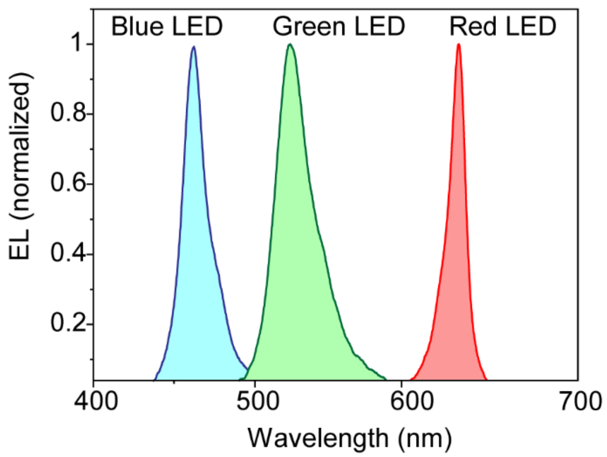

Supplementary Fig. 6 | Emission spectra of RGB channels emitted by the LC-10W diode. The data were experimentally measured utilizing a Thorlabs fiber optics spectrometer coupled via an optical fiber to the emitting surface of the LED.

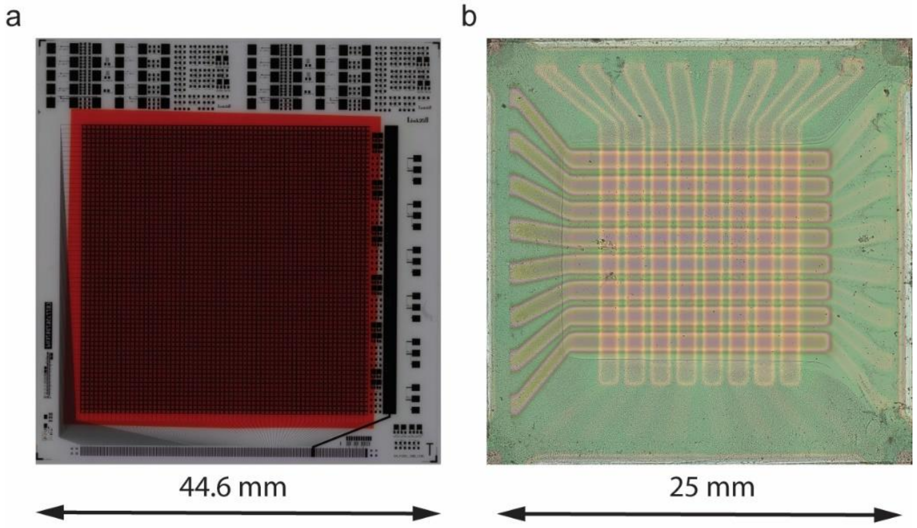

Supplementary Fig. 7 | Photos of a. TFT array (G-layer) and b. RGB stacked cross-bar array used in this study.

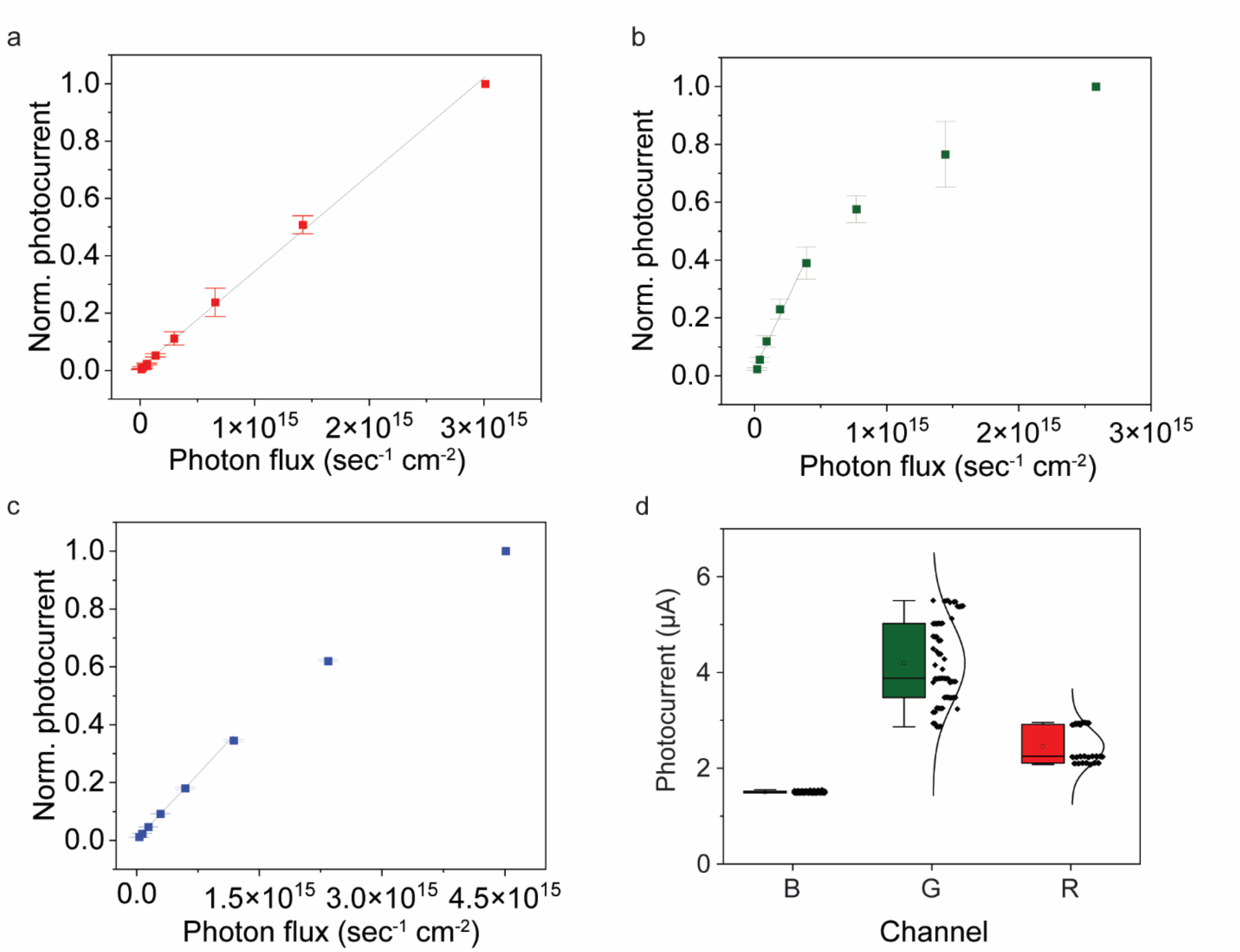

Supplementary Fig. 8 | Linearity of red (a), green (b), blue (c) channels at higher flux for the vertically stacked arrays. d. Statistics of blue, green, and red stacked channels photocurrents for a cross-bar array collected at $3 \cdot 10^{14}$ sec^{-1} cm^{-2} photon flux from blue, green, and red LEDs accordingly.

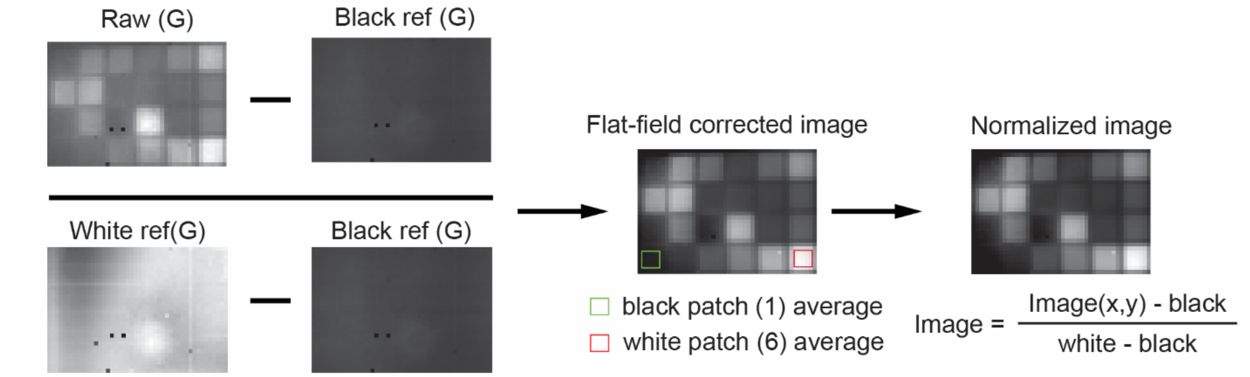

Supplementary Fig. 9 |TFT Image post-processing procedure. We obtained reference black and white images for flat-field correction by measuring white and matte-black paper. First, we subtracted the black image from both the raw and white images. Then, we divided the raw image by the white image to obtain the flat-field corrected image. Finally, the images were normalized by subtracting the average brightness of the black patch from a ColorChecker image and dividing by the average brightness of the white patch.

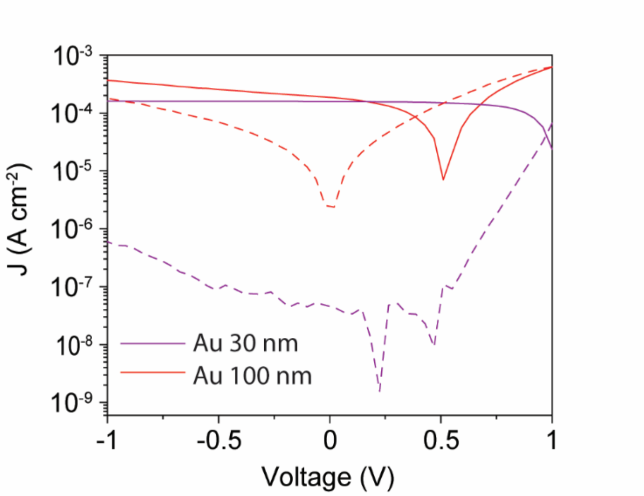

Supplementary Fig. 10 | Comparative performance of red detectors with 30 nm and 100 nm bottom gold electrode. The J-V (current-voltage) performance curves are derived from samples with detector structures identical to those of the red detectors, arranged in a stacked configuration.

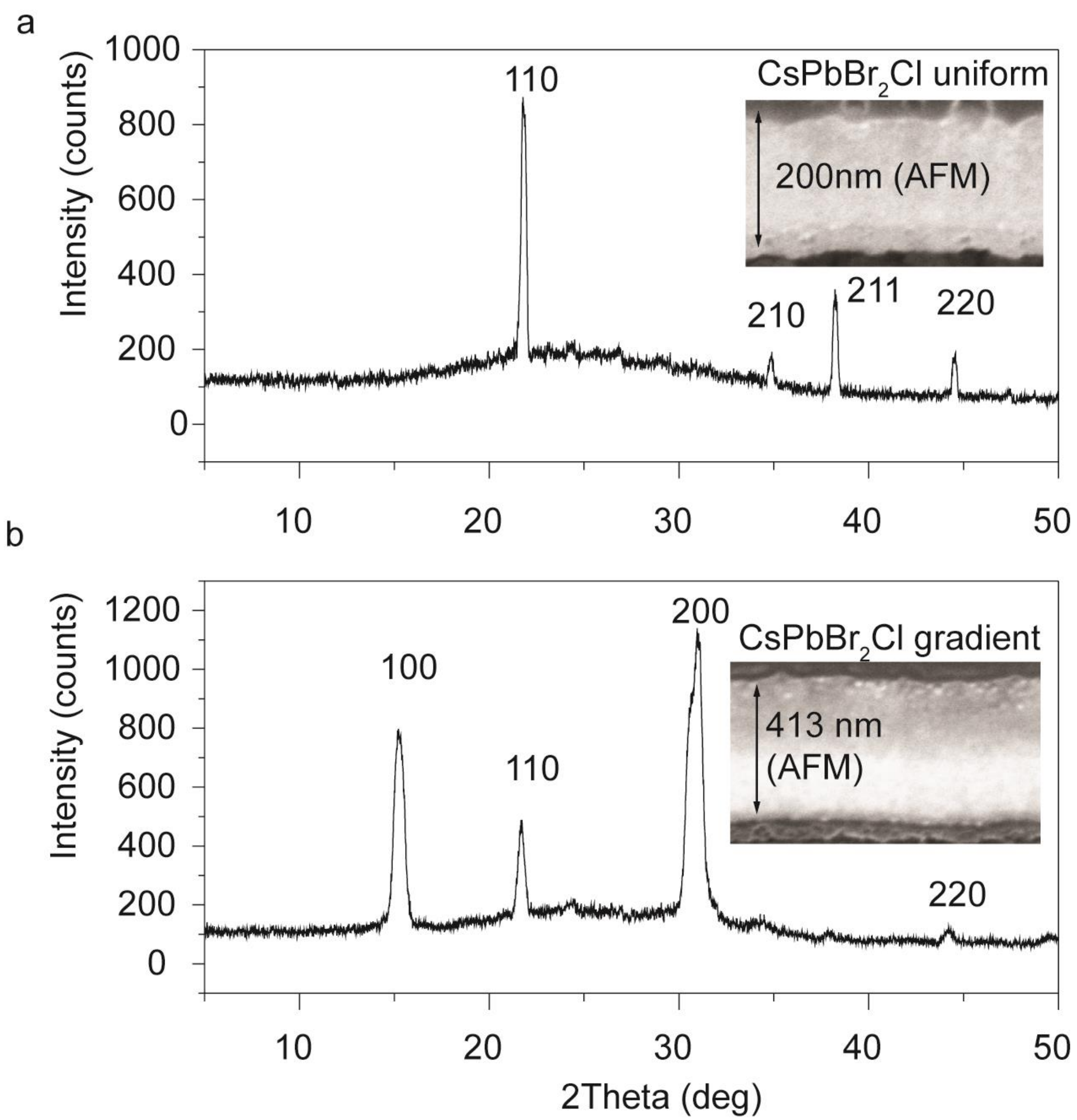

Supplementary Fig. 11 | X-ray diffraction patterns and SEM cross-section images of $CsPbBr_2Cl$ films. a. Uniformly co-evaporated $CsPbBr_2Cl$ and **b**. Gradient-grown $CsPbBr_2Cl$. Reflections are marked with corresponding (hkl) indices, assuming cubic structure for $CsPbBr_2Cl$.

Supplementary References

1 Ruiz-Ruiz, M. *et al.* Real-time microscopy of the relaxation of a glass. *Nat. Phys.* **19**, 1509-1515 (2023).

2 Cui, P. *et al.* Planar p–n homojunction perovskite solar cells with efficiency exceeding 21.3%. *Nat. Energy* **4**, 150-159 (2019).

3 Zhu, H. L. *et al.* Room-Temperature Solution-Processed NiOx:PbI2 Nanocomposite Structures for Realizing High-Performance Perovskite Photodetectors. *ACS Nano* **10**, 6808-6815 (2016).

4 Sutherland, B. R. *et al.* Sensitive, Fast, and Stable Perovskite Photodetectors Exploiting Interface Engineering. *ACS Photonics* **2**, 1117-1123 (2015).

5 Dou, L. *et al.* Solution-processed hybrid perovskite photodetectors with high detectivity. *Nat. Commun.* **5**, 5404 (2014).

6 *Thorlabs photodetectors webpage*, <https://www.thorlabs.com/newgrouppage9.cfm?objectgroup_id=285> (2024).

7 *Hamamatsu photodetectors webpage*, <https://www.hamamatsu.com/eu/en/product/optical-sensors/photodiodes/si-photodiodes/S12698-07.html> (2024).

8 Armin, A. *et al.* Narrowband light detection via internal quantum efficiency manipulation of organic photodiodes. *Nat. Commun.* **6**, 6343 (2015).

9 Xing, S. *et al.* Organic Thin-Film Red-Light Photodiodes with Tunable Spectral Response Via Selective Exciton Activation. *ACS Appl. Mater. Interfaces* **12**, 13061-13067 (2020).

10 Tang, Z. *et al.* Polymer:Fullerene Bimolecular Crystals for Near-Infrared Spectroscopic Photodetectors. *Adv. Mater.* **29**, 1702184 (2017).

11 Geisz, J. F. *et al.* Six-junction III–V solar cells with 47.1% conversion efficiency under 143 Suns concentration. *Nature Energy* **5**, 326-335 (2020).

12 Dimroth, F. *et al.* Four-Junction Wafer-Bonded Concentrator Solar Cells. *IEEE J. Photovolt.* **6**, 343-349 (2016).

13 Dimroth, F. *et al.* 3-6 junction photovoltaic cells for space and terrestrial concentrator applications. In *Conference Record of the Thirty-first IEEE Photovoltaic Specialists Conference, 2005.* 525-529.https://doi.org/10.1109/PVSC.2005.1488185

14 Chai, J. *et al.* High responsivity and high speed InGaN-based blue-light photodetectors on Si substrates. *RSC Adv* **11**, 25079-25083 (2021).

15 Liu, X. *et al.* High-Bandwidth InGaN Self-Powered Detector Arrays toward MIMO Visible Light Communication Based on Micro-LED Arrays. *ACS Photonics* **6**, 3186-3195 (2019).

16 Currie, M. *et al.* High-speed high-responsivity low temperature grown GaAs detector. In *IEEE Photonics Conference 2012.* 312-313.https://doi.org/10.1109/IPCon.2012.6358617

17 Helmers, H. *et al.* Advancing solar energy conversion efficiency to 47.6% and exploring the spectral versatility of III-V photonic power converters. In *SPIE Photonics West: Physics, Simulation, and Photonic Engineering of Photovoltaic Devices XII.* 1288103 (2024).https://doi.org/10.1117/12.3000352

18 Haggren, T. *et al.* Strain-Engineered Multilayer Epitaxial Lift-Off for Cost-Efficient III–V Photovoltaics and Optoelectronics. *ACS Appl. Mater. Interfaces* **15**, 1184-1191 (2023).

19 Dimroth, F. *et al.* Metamorphic GayIn1-yP/ Ga1-xInxAs tandem solar cells for space and for terrestrial concentrator applications at C>1000 suns. *Prog. Photovolt.: Res. Appl.* **9**, 165-178 (2001).
20 Park, K. W., Park, C. Y. & Lee, Y. T. Band gap tunability of molecular beam epitaxy grown lateral composition modulated GaInP structures by controlling V/III flux ratio. *Appl. Phys. Lett.* **101**, 051903 (2012).
21 Merrill, J. & Senft, D. C. Directions and materials challenges in high-performance photovoltaics. *JOM* **59**, 26-30 (2007).
22 Akimoto, K. *et al.* Photoluminescence killer center in AlGaAs grown by molecular-beam epitaxy. *J. Appl. Phys.* **59**, 2833-2836 (1986).
23 Parmar, M. & Reeves, S. J. Selection of Optimal Spectral Sensitivity Functions for Color Filter Arrays. In *2006 International Conference on Image Processing.* 1005-1008 (2006).https://doi.org/10.1109/ICIP.2006.312669
24 Wu, J. When group-III nitrides go infrared: New properties and perspectives. *J. Appl. Phys.* **106**, 011101 (2009).
25 Wu, J. *et al.* Unusual properties of the fundamental band gap of InN. *Appl. Phys. Lett.* **80**, 3967-3969 (2002).
26 Kour, R. *et al.* Recent advances and challenges in Indium Gallium Nitride (InxGa1-xN) materials for solid state lighting. *ECS J. Solid State Sci. Technol* **9** (2019).
27 Schley, P. *et al.* Dielectric function and van hove singularities for In-rich InxGa1-xN alloys: comparison of N- and metal-face materials. *Phys. Rev. B* **75**, 205204 (2007).
28 Jones, R. E. *et al.* Evidence for p-Type Doping of InN. *Phys. Rev. Lett.* **96**, 125505 (2006).
29 Ohsawa, J., Kozawa, T., Ishiguro, O. & Itoh, H. Selective Detection of Blue and Ultraviolet Light by An InGaN/GaN Schottky Barrier Photodiode. *Jpn. J. Appl. Phys.* **45**, L614 (2006).
30 Chiou, Y.-Z. *et al.* InGaN/GaN MQW p–n junction photodetectors. *Solid·State Electron.* **46**, 2227-2229 (2002).
31 Kang, C.-M. *et al.* Monolithic integration of AlGaInP-based red and InGaN-based green LEDs via adhesive bonding for multicolor emission. *Sci. Rep.* **7**, 10333 (2017).
32 Zhang, S., Wang, H., Wang, H. & Jiang, H. InGaN/GaN MQWs green-light photodetectors with thin GaN barrier layers. In *Applied Optics and Photonics China (AOPC2019).* 1133403 (SPIE, 2019).https://doi.org/http://dx.doi.org/10.1117/12.2539045
33 Ager, J. W. *et al.* InGaN/Si heterojunction tandem solar cells. In *2008 33rd IEEE Photovoltaic Specialists Conference.* 1-5 (IEEE, 2008). https://doi.org/10.1109/PVSC.2008.4922663
34 Vaisman, M. *et al.* Direct-Gap 2.1–2.2 eV AlInP Solar Cells on GaInAs/GaAs Metamorphic Buffers. *IEEE J. Photovolt.* **6**, 571-577 (2016).
35 Ben Slimane, A. *et al.* 1.73 eV AlGaAs/InGaP heterojunction solar cell grown by MBE with 18.7% efficiency. *Prog. Photovolt.* **28**, 393-402 (2020).
36 Zhao, S. *et al.* AlInP photodiode x-ray detectors. *J. Phys. D: Appl. Phys.* **52**, 225101 (2019).
37 Emanuel Thomet, J. *et al.* Bandgap engineering of indium gallium nitride layers grown by plasma-enhanced chemical vapor deposition. *J. Vac. Sci. Technol. A* **40** (2022).
38 Perlin, P. *et al.* Influence of pressure on the optical properties of InxGa1-xN epilayers and quantum structures. *Phys. Rev. B* **64**, 115319 (2001).

39 Azzellino, G. *et al.* Fully Inkjet-Printed Organic Photodetectors with High Quantum Yield. *Adv. Mater.* **25**, 6829-6833 (2013).

40 Lee, S.-H. *et al.* Toward color-selective printed organic photodetectors for high-resolution image sensors: From fundamentals to potential commercialization. *Materials Science and Engineering: R: Reports* **147**, 100660 (2022).

41 Zhao, T., Xia, K., Natali, D. & Pecunia, V. Solution-Based Integration of Vertically Stacked Organic Photodetectors Toward Easy-To-Fabricate Filterless Multi-Color Light Sensors. *Adv. Opt. Mater.* **10**, 2200862 (2022).

42 Baierl, D. *et al.* A hybrid CMOS-imager with a solution-processable polymer as photoactive layer. *Nat. Commun.* **3**, 1175 (2012).

43 Wu, J.-L. *et al.* Top-Illuminated Organic Photodetectors beyond 1000 nm Wavelength Response Enabled by a Well-Defined Interfacial Engineering. *Adv. Opt. Mater.* **10**, 2101723 (2022).

44 Suemori, K. Assessment of sputtering damage in organic layer surface based on energy distribution of positively charged particles formed during facing-target sputtering of indium–tin oxide. *Org. Electron.* **116**, 106764 (2023).

45 Kim, D.-H. *et al.* A high performance semitransparent organic photodetector with green color selectivity. *Appl. Phys. Lett.* **105** (2014).

46 Liu, H. *et al.* Orthogonal solvent-sequential deposition of a nonfullerene acceptor solution on polymer donor film: complete interpenetration and highly efficient inverted organic solar cells. *J. Mater. Chem. A* **11**, 19860-19869 (2023).

47 Gaikwad, A. M. *et al.* Identifying orthogonal solvents for solution processed organic transistors. *Org. Electron.* **30**, 18-29 (2016).

48 Kovacik, P., Assender, H. E. & Watt, A. A. R. Morphology control in co-evaporated bulk heterojunction solar cells. *Sol. Energy Mater. Sol. Cells* **117**, 22-28 (2013).

49 Riede, M. *et al.* Efficient Organic Tandem Solar Cells based on Small Molecules. *Adv. Funct. Mater.* **21**, 3019-3028 (2011).

50 Zhang, T. *et al.* High-Performance Filterless Blue Narrowband Organic Photodetectors. *Adv. Funct. Mater.* **34**, 2308719 (2024).

51 Lim, S.-J. *et al.* Organic-on-silicon complementary metal–oxide–semiconductor colour image sensors. *Sci. Rep.* **5**, 7708 (2015).

52 Shekhar, H. *et al.* Hybrid image sensor of small molecule organic photodiode on CMOS – Integration and characterization. *Sci. Rep.* **10**, 7594 (2020).

53 Bulliard, X. *et al.* Dipolar donor–acceptor molecules in the cyanine limit for high efficiency green-light-selective organic photodiodes. *J. Mater. Chem. C* **4**, 1117-1125 (2016).

54 Aihara, S. *et al.* Stacked Image Sensor With Green- and Red-Sensitive Organic Photoconductive Films Applying Zinc Oxide Thin-Film Transistors to a Signal Readout Circuit. *IEEE Trans. Electron Devices* **56**, 2570-2576 (2009).

55 Sakai, T. *et al.* Color-Filter-Free Three-Layer-Stacked Image Sensor Using Blue/Green-Selective Organic Photoconductive Films with Thin-Film Transistor Circuits on CMOS Image Sensors. *ACS Appl. Electron. Mater.* **3**, 3085-3095 (2021).

56 Park, S. *et al.* Transparent organic photodiodes for high-detectivity CMOS image sensors. *Optica* **9**, 992-999 (2022).

57 Takagi, T. *et al.* Image Sensor with Organic Photoconductive Films by Stacking Red/Green and Blue Components. *Electron. Imaging* **28**, 1-4 (2016).
58 Nasrollahi, B., Jailani, J. M., Zhao, T. & Pecunia, V. Printable organic photodetectors with gain toward high-performance vertically stacked color sensors. *IEEE Journal on Flexible Electronics* **2**, 285-292 (2023).
59 Lim, Y. *et al.* Green-Light-Selective Organic Photodiodes with High Detectivity for CMOS Color Image Sensors. *ACS Appl. Mater. Interfaces* **12**, 51688-51698 (2020).
60 Guo, H. *et al.* Unsymmetric squaraine for narrow band green-selective organic photodetectors. *Org. Electron.* **92**, 106122 (2021).
61 Lee, G. H. *et al.* Green-light-selective organic photodiodes for full-color imaging. *Opt. Express* **27**, 25410-25419 (2019).
62 Li, N., Eedugurala, N., Azoulay, J. D. & Ng, T. N. A filterless organic photodetector electrically switchable between visible and infrared detection. *Cell Rep. Phys. Sci.* **3** (2022).
63 Liu, J. *et al.* Challenges and recent advances in photodiodes-based organic photodetectors. *Phys. Chem. Chem. Phys.* **51**, 475-503 (2021).
64 Li, W. *et al.* Squarylium and rubrene based filterless narrowband photodetectors for an all-organic two-channel visible light communication system. *Org. Electron.* **37**, 346-351 (2016).
65 Lu, J. H. *et al.* High-Performance organic photodiodes for Blue-Light hazard detection. *Chem. Eng. J.* **437**, 135327 (2022).
66 Seo, H. *et al.* Color Sensors with Three Vertically Stacked Organic Photodetectors. *Jpn. J. Appl. Phys.* **46**, L1240 (2007).
67 Fukuda, T. *et al.* Improved optical-to-electrical conversion efficiency by doping silole derivative with low ionization potential. *Phys. Status Solidi A* **209**, 2324-2329 (2012).
68 Aydin, E. *et al.* Sputtered transparent electrodes for optoelectronic devices: Induced damage and mitigation strategies. *Matter* **4**, 3549-3584 (2021).
69 Ellmer, K. & Welzel, T. Reactive magnetron sputtering of transparent conductive oxide thin films: Role of energetic particle (ion) bombardment. *J. Mater. Res.* **27**, 765-779 (2012).
70 Reddy, S. H. *et al.* Holistic Approach toward a Damage-Less Sputtered Indium Tin Oxide Barrier Layer for High-Stability Inverted Perovskite Solar Cells and Modules. *ACS Appl. Mater. Interfaces* **14**, 51438-51448 (2022).
71 Liu, K. *et al.* Reducing sputter induced stress and damage for efficient perovskite/silicon tandem solar cells. *J. Mater. Chem. A* **10**, 1343-1349 (2022).
72 Schubert, S. *et al.* Eclipse Pulsed Laser Deposition for Damage-Free Preparation of Transparent ZnO Electrodes on Top of Organic Solar Cells. *Adv. Funct. Mater.* **25**, 4321-4327 (2015).
73 Sakhatskyi, K. *et al.* Assessing the drawbacks and benefits of ion migration in lead halide perovskites. *ACS Energy Letters* **7**, 3401-3414 (2022).
74 Hung, P.-C. Colorimetric calibration in electronic imaging devices using a look-up-table model and interpolations. *Journal of Electronic Imaging* **2** (1993).
75 Tominaga, S. *Color control using neural networks and its application*. Vol. 2658 EI (SPIE, 1996).
76 http://www.brucelindbloom.com/ (2025).
